# Supplementary material for: Satisfactory In Vitro Activity of Ceftolozane–Tazobactam against Carbapenem-Resistant Pseudomonas aeruginosa But Not against Klebsiella pneumoniae Isolates
Source: Medicina (Kaunas). 2023 Mar 7;59(3):518. doi: 10.3390/medicina59030518 (PMC10057464; doi:10.3390/medicina59030518)
Supplement: Supplementary file 1 [file medicina-59-00518-s001.zip › medicina-2247277-supplementary.pdf]

Supplementary material

Satisfactory *in vitro* activity of ceftolozane-tazobactam against carbapenem-resistant *Pseudomonas aeruginosa* but not against *Klebsiella pneumoniae* isolates

Alicja Sękowska[1,2]*, Marta Grabowska[1,3], Tomasz Bogiel[1,2]*

[1] Microbiology Department, Ludwik Rydygier Collegium Medicum in Bydgoszcz, Nicolaus Copernicus University in Torun, Poland

[2] Clinical Microbiology Department dr Antoni Jurasz University Hospital No 1 in Bydgoszcz, Poland

[3] dr Jan Biziel University Hospital No 2 in Bydgoszcz, Poland

* Correspondence: Alicja Sękowska (e-mail: asekowska@cm.umk.pl), Tomasz Bogiel (t.bogiel@cm.umk.pl), Microbiology Department, Ludwik Rydygier Collegium Medicum in Bydgoszcz, Nicolaus Copernicus University in Torun, 9 Maria Skłodowska-Curie St, 85-094 Bydgoszcz, Poland, tel.: +48 52 585 44 80

Table S1. The detailed susceptibility profiles of the examined *P. aeruginosa* strains (n = 150)

Patient No./isolate	Piperacillin/tazobactam	Ceftazidime	Cefepime	Imipenem	Meropenem	Tobramycin	Amikacin	Ciprofloxacin	Levofloxacin	Colistin	Ceftolozane/tazobactam	Ceftolozane/tazobactam MIC
1	S	S	R	R	R	R	R	R	R	S	S	2
2	R	R	R	R	R	S	R	R	R	S	S	2
3	R	R	R	R	S	R	R	R	R	S	R	8
4a	R	R	S	R	S	S	S	R	R	S	S	0.5
4b	S	S	R	R	S	R	S	S	R	S	S	0.75
5	S	S	S	R	R	S	S	R	R	S	S	1
6	R	R	R	R	R	S	S	R	R	S	S	0.75
7	R	R	R	R	R	R	S	R	R	S	S	1.5
8	R	R	R	R	R	S	S	R	S	S	S	2
9	R	R	I	R	S	R	S	R	R	S	S	0.5
10a	R	R	R	R	R		S	R	R	S	S	0.5
10b	R	S	S	R	R	S	S	R	R	S	S	1
11a	R	R	R	R	R	S	S	R	R	S	S	1
11b	R	R	S	R	R	R	S	R	R	S	S	0.75
12	R	S	R	R	R	S	S	S	R		S	1
13	R	R	R	R	R	R	R	R	R	S	S	1
14a	R	R	R	R	R	S	S	S	S	S	S	0.75
14b	R	R	R	R	R	S	S	S	S	S	S	2
15	R	R	R	R	R	S	S	S	S	S	S	1.5

16	R	R	R	R	R	R	R	R	R	S	S	>256
17	R	R	R	R	S	S	S	S		S	S	1
18	R	R	R	R	R	R	R	R	R	S	R	6
19	S	S	S	R	S	S	S	S	S	S	S	1
20a	R		R	R	R	S	S	R	R	S	S	4
20b	R	R	I	R	R	S	S	R	R	S	R	6
20c	R	R	R	R	R	R	R	R	R	S	S	3
20d	R	R	R	R	R	R	R	S	S	S	S	4
20e	R	R	R	R	S	R	R	S	S	S	S	3
20f	R	R	R	R	S	S	S	S	S	S	S	2
20g	S	S	S	R	S	S	S	S	S	S	S	1.5
21a	R	I	R	R	R	R	R	R	R	R	S	1.5
21b	R	I	R	R	R	R	R	R	R	S	S	3
22	R	R	R	R	R	S	S	S	R	S	S	1
23	R	R	I	R	R	S	S	I	I	S	R	>256
24	R	R	R	R	R	S	S	I	I	S	S	1.5
25	R	R	R	R	R	S	S	R	R	S	S	1.5
26	S	S	S	R	S	S	S	S		S	S	0.5
27a	R	R	R	R	R	S	S	R	R	S	S	1
27b	I	R	R	R	R	S	S	R	I	S	S	4
28	R	R	R	R	R	S	S	R	R	S	S	2
29	R	R	R	R	R	S	S	R	R	S	S	2
30	R	R	R	R	R	S	S	R	R	S	S	4
31	I	I	I	R	I	S	S	R	R	S	S	4
32	R	R	R	R	R	S	S	R	R	S	S	1.5
33	S	S	S	R	R	R	R	R	R	S	S	1.5
34	R	R	R	R	R	S	S	S	R	S	S	3
35	R	R	R	R	R	S	S	S	S	S	S	2
36	R	R	R	R	R	S	S	R	R	S	S	3
37	R	R	R	R	R	S	S	S	R	S	S	1.5
38	R	R	R	R	R	S	S	R	R	S	S	1.5
39	S	S	S	R	S	S	S	S	S	S	S	1.5
40	S	S	S	R	R		R	R	R		S	1.5
41	S	S	S	I	R	S	S	S	R	S	S	1
42	R	R	R	R	R	R	R	R	R	S	R	96
43	R	R	R	R	R	S	S	R	R	S	S	2
44	R	R	R	R	S	S	S	S	S	S	S	4
45	I	I	I	R	S	S	S	R	R	S	S	0.5
46	S	S	S	R	S	S	S	S	S	S	S	1.5
47	R	R	R	R	R	R	R	S	S		R	256
48	S	S	S	R	S	S	S	S	R	S	S	0.75
49	R	R	R	R	R	S	S	R	R	S	S	2
50a	R	R	R	R	R	S	S	R	R	S	S	3
50b	R	R	R	R	R	S	S	I	R	S	R	6
51	R	S	R	I	R	R	R	R	R	S	S	2
52	S	R	R	I	R	S	R	R	R	S	S	2
53	R	R	R	R	R	S	S	S	S	S	S	1.5

54	R	R	R	R	R	R	S	R	R	S	R	>256
55	R	R	R	R	R	S	R	R	R	S	S	4
56	R	R	R	R	R	R	R	S	S	S	R	>256
57	R	R	R	R	R	S	S	S	S	S	S	2
58	S	S	S	R	S	S	S	S	S		S	0.5
59	I	R	R	R	I	R	R	R	R	R	R	8
60a	R	R	R	R	S	S	S	R	R	R	R	6
60b	S	S	S	R	S	S	S	R	R	S	S	0.75
61	R	R	R	R	R	S	S	S	S	S	S	1
62	R	R	R	R	R	R	R	S	R		S	1
63a	R	S	R	I	S	R	R	S	S	S	S	1.5
63b	S	S	S	S	S	R	S	S	S	S	S	0.25
64	R	R	R	R	S	S	S	S	S	S	S	1
65	R	R	R	R	S	S	S	S	R	S	S	1.5
66a	R	R	R	R	R	S	S	R	R	R	S	3
66b	R	R	S	R	R	S	S	R	R	R	S	1.5
67	S	S	R	R	R	S	S	S	R		S	0.75
68	R	R	R	S	S	R	R	R	R	S	S	1
69	R	R	R	R	R	S	S	R	R	S	R	6
70	R	R	R	I	S	S	S	R	I	S	S	1.5
71a	R	R	R	R	R	S	S	R	R	S	S	4
71b	R	S	R	R	R	S	S	S	R	S	S	1.5
72	R	R	R	R	R	R	R	R	R	S	R	>256
73	R	R	R	R	R	R	R	R	R	S	S	0.75
74	R	R	R	R	R	S	S	R	R	S	R	8
75	S	S	S	R	R	S	S	S	S	S	S	1
76	R	S	R	R	S	S	S	R	R	S	S	0.75
77	S	R	S	R	R	R	R	R	R	S	S	1
78	R	R	S	R	R	R	R	S		S	S	4
79	R	R	S	R	R	R	R	R	R	S	S	0.75
80	R	R	R	R	R	S	S	S	S	S	S	2
81	R	R	S	R	R	R	S	R	R	S	R	>256
82	S	S	S	R	R	S	S	R	R	S	S	0.75
83	R	I	R	R	R	R	S	R	R	S	S	0.75
84	R	R	R	R	R	R	R	R	I	S	R	>256
85	R	R	R	R	R	S	S	R	R	S	S	4
86	R	R	R	R	R	R	R	R	R	S	R	>256
87	R	R	R	R	R	R	R	R	R	S	S	1
88	R	R	R	R	R	R	R	R	R	S	S	1.5
89	R	R	R	R	R	R	R	R	R	S	S	1.5
90a	R	R	R	R	R	S	S	S	S	S	S	0.75
90b	R	R	R	R	R	S	S	S	S	S	S	2
90c	S	S	S	R	S	S	S	S	S	S	S	2
91	R	R	R	R	R	S	S	R	R	S	S	1
92	R	I	R	R	R	S	S	R	R	S	S	2
93a	R	R	R	R	R	S	S	R	R	S	S	1.5
93b	R	R	R	R	R	S	S	S	R	R	S	2

94	S	S	S	R	R	S	S	S	S		S	1
95	S	S	S	R	S	S	S	S	S		S	1
96	S	S	S	R	S	S	S	S	S	S	S	2
97	R	R	R	R	S	S	S	S	I	S	S	1
98	R	R	R	R	R	S	S	S	R	S	R	48
99	R	R	R	R	R	S	S	R	R	S	S	2
100a	I	R	R	R	R	S	S	R	R	R	S	0.19
100b	R	R	R	R	R	S	S	R	R	S	S	2
101	R	R	R	R	R	S	S	R	R	S	S	1.5
102	S	S	S	R	S	S	S	S	S	S	S	1
103a	R	R	R	R	R	S	S	S	R	S	S	1.5
103b	R	R	R	R	R		R	R		S	S	0.75
104	S	S	S	S	S	S	S	S	S	S	S	0.75
105	R	R	R	R	R	S	S	R	R	R	S	3
106	S	R	S	R	R	R	R	S	S	S	S	0.75
107	R	S	R	R	R	S	R	R	R	S	S	1.5
108	R	R	R	R	R	S	S	R	R	S	S	1
109	S	S	S	R	S	S	S	S		S	S	0.25
110	R	R	R	R	S	S	S	S		S	R	256
111	R	R	R	R	S	R	R	S	R	S	S	1
112a	R	R	R	R	R	S	S	R	R	R	S	4
112b	R	R	R	R	R	S	R	R	R	S	S	4
112c	R	R	R	R	R	S	S	R	R	S	R	8
113	R	R	S	R	R	S	S	S	S	S	S	0.75
114	R	R	R	R	R	R	R	R	R	S	R	256
115	R	R	R	R	S	R	R	R	R	R	R	64
116	R	R	S	R	R	R	R	R	R	S	S	1.5
117	R	R	R	R	R	S	S	R	R		S	1.5
118	R	R	R	R	S	S	S	S	S	S	S	4
119	R	R	R	R	S	S	S	R	R	S	S	1
120	R	R	R	R	S	R	R	R	R	S	R	6
121	S	S	S	R	S	S	S	S	S	S	S	0.75
122	R	R	R	R	R	R	R	R	I	S	R	>256
123	I	I	I	R	S	S	S	R	R	S	S	0.75
124	R	S	R	S	S	R	R	R	R	S	S	2
125	R	R	S	R	R	R	R	R	R	S	S	4
126	R	R	R	R	R	S	S	R	R	S	S	2

a - g - subsequent isolates derived from the same patient; EUCAST antimicrobials susceptibility categories: I - susceptible, increased exposure, R - resistant, S - susceptible at standard doses; MIC – minimal inhibitory concentration

Table S2. The detailed susceptibility profiles of the examined *K. pneumoniae* strains (*n* = 100)

Patient No./isolate	Piperacillin/tazobactam	Cefotaxime	Ceftazidime	Cefepime	Imipenem	Meropenem	Ertapenem	Tobramycin	Gentamicin	Amikacin	Ciprofloxacin	Levofloxacin	Trimethoprim/sulfamethoxazole	Colistin	ESBL	Ceftolozane/tazobactam	Ceftolozane/tazobactam MIC
1	R	R	R	R	I	I	R	R	S	I	R	R	R	S	+	R	>256
2a	R	R	R	R	S	S	R	R	I	S	R	R	R	S	+	R	48
2b	R	R	R	R	S	R	R	S	S	S	I	I	S	S	+	R	256
3	R	R	R	R	R	R	R	R	R	R	R	R			+	R	256
4	S	R	R	R	S	R	R	R	R	S	R	R	R	R	+	S	0.75
5a	R	R	R	R	R	R	R	I	S	I	S	S	R	S	+	R	>256
5b	R	R	R	R	R	R	R	S	S	S	S	S	R	S	+	R	>256
6	R	R	R	R	I	R	R	R	S	R	R	R	R	R	+	R	256
7	R	R	R	R	S	S	R	R	R	S	R	R	R		+	R	6
8	R	R	R	R	S	I	R	S	S	S	R	R	R	S	+	R	>256
9	R	R	R	R	I	R	R	R	R	I	R	R	R	R	+	S	2
10	R	R	R	R	I	R	R	R	R	I	R	R	R	S	+	R	4
11	R	R	R	S	R	R	R	R	R	I	R	R	R		+	S	1
12	R	R	R	S	R	R	R	R	R	I	R	R	R		+	R	4
13	R	R	R	R	I	I	R	R	R	R	S	R	R	S	+	S	1
14					R	R	R								+	R	>256
15	R	R	R	R	I	R	R	R	R	R	R	R			+	S	0.5
16	R	R	R	R	S	R	R	R	R	S	R	R	R	S	+	R	>256
17	S	S	S	S	S	S	R	S	S	S	R	R	R	S	+	S	0.38
18	R	R	R	R	S	S	R	R	R	I	R	R	R	S	+	R	48
19	R	R	I	R	I	R	R	R	R	R	S	R	R	R	+	S	0.38
20	R	R	R	R	I	R	R	R	R	S	R	R	R	R	+	R	>256
21a	I	R	R	R	S	I	S	R	S	I	R		R		+	S	1
21b	R	R	R	R	I	R	R	R	S	I	R	R	R	S	+	R	>256
22	R	R	R	R	I	R	R	R	R	S	R	R	R		+	R	>256
23	R	R	R	R	S	S	R	R	I	S	S	S	R	S	+	R	48
24	R	R	R	R	I	I	R	R	R	R	S	R		S	+	R	6
25	R	R	R	R	S	I	S	R	S	I	R	R	R	S	+	S	1.5
26	R	R	R	R	S	I	R	R	S	I	R	R	R	S	+	R	6
27	S	R	R	R	R	I	R	S	S	S	R	R	R	R	+	R	>256
28	R	R	R	R	R	R	R	R	R	R	R	R		S	+	S	2
29	I	R	R	R	I	I	R	R	S	R	R	R	S	S	+	R	6
30	R	R	R	R	S	S	R	R	I	S	R	R	R	S	+	S	2
31a	R	R	R	R	R	R	R	R	R	S	R	R	R	R	+	R	256
31b	R	R	R	R	S	I	R	R	R	S	R	R	R	R	+	R	>256

32	R	R	R	R	S	R	R	R	R	R	R	R	S	S	+	R	256
33	S	R	R	R	S	S	R	R	S	I	R	R	R		+	S	0.75
34	R	R	R	R	S	S	R	R	I	S	R	R	R		+	R	4
35a	R	R	R	R	S	S	R	R	S	R	R	R	S	R	+	R	4
35b	I	R	R	R	S	S	R	R	S	R	R	R	S	R	+	R	6
36	R	R	R	R	I	R	R	R	R	S	R	R	R	S	+	R	>256
37	R	R	R	R	R	R	R	R	S	I	R		R	S	+	R	256
38	R	R	R	R	I	R	R	R	R	R	R	R			+	R	16
39	R	R	R	R	R	R	R	S	S	S	R	R	R	R	+	R	256
40	R	R	R	R	R	R	R	S	S	S	R	R	R	S	+	R	>256
41	R	R	R	R	S	S	R	R	I	S	S	S	R		+	S	1
42	R	R	R	R	S	I	R	R	S	R	R	R	R	S	+	R	>256
43	R	R	R	R	R		R	R	R	R	R	R			+	S	0.5
44	R	R	R	R	S	S	R	R	R	I	R		R	R	+	R	96
45	R	R	R	R	R	R	R	R	I	S	R	R	R	S	+	R	>256
46	R	R	R	R	S	I	R	R	R	S	R	R	R	S	+	R	>256
47	R	R	R	R	R	O	R	R	R	R	R	R		S	+	S	0.38
48	R	R	R	R	S	S	R	R	R	S	R	R	R		+	R	48
49	R	R	R	R	S	S	R	R	I	S	R	R	R	S	+	R	>256
50	R	R	R	R	R	R	R	R	R	I	R	R	R	R	+	S	0.75
51	R	R	R	R	R	R	R	S	S	S	R	R	R	S	+	R	>256
52	R	R	R	R	S	S	R	R	I	S	R	R	R		+	S	1
53	R	R	R	R	S	I	R	R	R	S	R	R	R	S	+	R	>256
54	R	R	R	R	S	S	R	R	R	R	R	R	R	R	+	R	4
55	R	R	R	R	S	S	R	R	S	I	R	R	R	S	+	R	>256
56	R	R	R	R	R	I	R	R	*S*	I	I	S	R	R	+	S	2
57	R	R	R	R	I	R	R	R	S	I	R	R	R	R	+	R	>256
58	R	R	R	S	R	R	R	R	R	R	R	R	R	R	+	R	3
59	R	R	R	R	R	R	R	R	S	I	R	R	S	S	+	R	256
60	R	R	R	R	S	S	R	R	R	S	R	R	R	S	+	S	0.75
61	R	R	R	S	R	R	R	R	S	R	R	R	R		+	R	3
62	R	R	R	R	R	R	R	R	R	I	R	R	R	R	+	S	1
63	R	R	R	R	I	R	R	S	S	S	R	R	R	S	+	R	>256
64	R	R	R	R	I	R	R	R	I	R	R	R	R	S	+	R	>256
65	R	R	R	R	R	R	R	R	R	I	R	R		S	+	S	2
66	R	R	R	R	R	R	R	R	S	I	R	R	R	S	+	S	0.38
67	R	R	R	R	I	I	R	R	S	I	R	R	R	S	+	R	>256
68	R	R	R	R	S	S	R	R	S	I	R	R	R	S	+	R	>256
69	R	R	R	R	S	S	R	R	R	I	R	R	R	R	+	R	4
70	R	R	R	R	S	S	R	R	I	S	R	R	R	S	+	R	196
71	R	R	R	R	S	R	R	R	R	R	R	R	R	R	+	R	256
72	R	R	R	R	R	R	R	R	S	R	I	R		S	+	S	0.5
73	R	R	R	R	S	I	R	R	I	S	R	R	R	S	+	R	256
74	R	R	R	R	S	S	R	R	R	I	R		R	R	+	R	3
75	R	R	R	R	S	S	R	R	I	S	R	R	R		+	R	12
76	R	R	R	R	I	I	R	R	R	I	R	R	R	S	+	R	256
77	R	R	R	R	S	R	R	R	R	R	R	R			+	R	4

78a	S	R	R	R	S	S	R	R	S	I	R	R	R	S	+	S	0.38
78b	I		R	R	S	S	R		S	S	R		R	S	+	S	0.5
79	R	R	R	R	S	S	R	R	I	S	R	R	R	S	+	R	>256
80	R	R	R	R	R	R	R	R	I	S	R	R	S	R	+	R	256
81	R	R	R	R	I	R	R	R	R	S	R	R	R	S	+	R	64
82	R	R	R	S	S	R	R	R	R	I	R	R	O		+	S	1.5
83	R	R	R	R	R	R	R	R	R	R	R	R	O	S	+	S	1.5
84	R	R	R	S	S	R	R	R	R	I	S	R	O	S	+	S	1.5
85a	R	R	R	R	I	S	S	R	S	S	R	R	O	R	+	S	2
85b	R	R	R	R	I	I	R	R	R	S	R	R	O	R	+	R	>256
86	R	R	R	R	S	I	S	R	I	S	R		O	S	+	R	6
87	R	R	R	R	I	I	R	R	I	S	R	R	O	R	+	R	256
88	R	R	R	R	S	S	R	R	R	S	R	R	O		+	S	1
89	R	R	R	R	S	S	R	R	I	S	R	R	O	S	+	R	>256
90	R	R	R	R	S	S	R	R	I	I	R		O	S	+	R	4
91	R	R	R	R	R	R	R	S	S	S	R	R	O	R	+	R	>256
92	R	R	R	R	R	R	R	R	S	S	R	R	O	R	+	R	8
93	R	R	R	R	S	R	S	R	I	S	S	S	O		+	R	12

a - b - subsequent isolates derived from the same patient; EUCAST antimicrobials susceptibility categories: I - susceptible, increased exposure, R - resistant, S - susceptible at standard doses; (+) - ESBL - extended spectrum beta-lactamases producers; MIC – minimal inhibitory concentration

Table S3. The origin of *P. aeruginosa* clinical strains included into the study (*n* = 206)

**Hospital unit/department/clinic**	***n***	**%**
Intensive Care	110	53.4
Pediatric Hematology and Oncology	38	18.4
Transplantation and General Surgery	23	11.2
Cardiology	11	5.3
Nefrology	9	4.4
Neurosurgery	5	2.4
Vascular Surgery and Angiology	4	1.9
Dermatology	3	1.5
Urology	3	1.5
Total	206	100%

**Specimen type**	***n***	**%**
Bronchoalveolar lavage fluid	69	33.5
Blood	56	27.2
Wound and bedsore swabs	33	16.0
Skin, catheter implantation site and surgery site swabs	13	6.3
Urine/catheterized urine	10	4.8
Stool	5	2.4
Ear swab	3	1.5
Fluid from the body cavities	3	1.5
Purulent material	2	1.0
Vascular catheter	2	1.0
The rest: peritoneal swab – 1, abdominal fluid – 1, sputum – 1, prosthesis – 1, peritoneal fluid – 1, bile – 1, pleural fluid – 1, rectal swab – 1, pharyngeal swab – 1, drain – 1	10	4.8
Total	206	100%

Table S4. The origin of *K. pneumoniae* clinical strains included into the study (*n* = 110)

**Hospital unit/department/clinic**	***n***	**%**
Intensive Care	51	46.5
Pediatric Hematology and Oncology	18	16.5
Transplantation and General Surgery	11	10.0
Nefrology	6	5.4
Urology with outpatients	5	4.5
Cardiology	5	4.5
Vascular Surgery and Angiology	4	3.6
Geriatrics	3	2.7
Neurology	2	1.8
The rest: Rehabilitation - 1, Endocrinology - 1, Cardiosurgery - 1, Pediatrics Allergology and Gastroenterology - 1, Infectious Diseases Hospital - 1	5	4.5
Total	110	100%

**Specimen type**	***n***	**%**
Urine/catheterized urine	22	20.0
Blood	21	19.1
Wound and bedsore swabs	17	15.5
Bronchoalveolar lavage fluid	17	15.5
Stool	6	5.4
Rectal swab	6	5.4
Vascular catheter	5	4.5
Skin, catheter implantation site and surgery site swabs	3	2.7
Purulent material	3	2.7
Pharyngeal swab	2	1.8
The rest: bile – 1, peritoneal swab – 1, tissue – 1, peritoneal fluid – 1, drainage fluid – 1, vaginal swab – 1, fluid from the cyst – 1, abdominal fluid – 1	8	7.4
Total	110	100%
